# 2-Phenyl­naphtho­[1,8-*de*][1,3,2]diaza­borinane

**DOI:** 10.1107/S1600536811026985

**Published:** 2011-07-09

**Authors:** Cathryn A. Slabber, Craig Grimmer, Matthew P. Akerman, Ross S. Robinson

**Affiliations:** a Warren Research Laboratory, School of Chemistry, University of KwaZulu-Natal, Private Bag X01, Scottsville, Pietermaritzburg 3209, South Africa

## Abstract

The title compound, C_16_H_13_BN_2_, is one compound in a series of diaza­borinanes featuring substitution at the 1, 2 and 3 positions in the nitro­gen–boron heterocycle. The title compound is slightly distorted from planarity, with a dihedral angle of 9.0 (5)° between the mean planes of the naphthalene system and the benzene ring. The *m*-carbon atom of the benzene ring exhibits the greatest deviation of 0.164 (2) Å from the 19-atom mean plane defined by all non-H atoms. The two N—B—C—C torsion angles are 6.0 (3) and 5.6 (3)°. In the crystal, mol­ecules are linked by π–π inter­actions into columns, with a distance of 3.92 (3) Å between the naphthalene ring centroids. Adjacent π-stacked columns, co-linear with the *b*-axis, are linked by C—H⋯π inter­actions.

## Related literature

For the synthesis, see: Letsinger & Hamilton (1958 ▶); Pailer & Fenzl (1961 ▶); Kaupp *et al.* (2003 ▶); Slabber (2011 ▶). For related structures and luminescence studies, see: Weber *et al.* (2009 ▶). Changes in illuminated volume were kept to a minimum, and were taken into account (Görbitz, 1999 ▶) by the multi-scan inter-frame scaling (*DENZO*/*SCALEPACK*; Otwinowski & Minor, 1997 ▶).
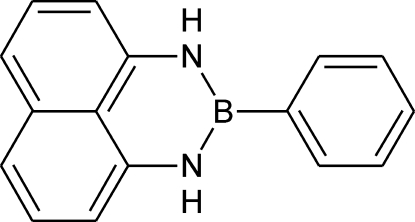


## Experimental

### 

#### Crystal data

C_16_H_13_BN_2_
*M* *_r_* = 244.10
Monoclinic, 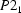

*a* = 11.0117 (7) Å
*b* = 5.4299 (2) Å
*c* = 11.7454 (7) Å
β = 117.574 (8)°
*V* = 622.52 (7) Å^3^
*Z* = 2
Mo *K*α radiation
μ = 0.08 mm^−1^
*T* = 298 K
0.45 × 0.35 × 0.35 mm

#### Data collection

Oxford Diffraction Xcalibur 2 CCD diffractometer
Absorption correction: multi-scan (*CrysAlis RED*; Oxford Diffraction, 2002 ▶) *T*_min_ = 0.966, *T*_max_ = 0.974
6492 measured reflections
2177 independent reflections
1601 reflections with *I* > 2σ(*I*)
*R*_int_ = 0.025

#### Refinement

*R*[*F* ^2^ > 2σ(*F* ^2^)] = 0.039
*wR*(*F* ^2^) = 0.121
*S* = 0.91
2173 reflections
173 parameters
1 restraint
H-atom parameters constrained
Δρ_max_ = 0.20 e Å^−3^
Δρ_min_ = −0.16 e Å^−3^

### 

Data collection: *CrysAlis CCD* (Oxford Diffraction, 2002 ▶5); cell refinement: *CrysAlis RED* (Oxford Diffraction, 2002 ▶); data reduction: *CrysAlis RED*; program(s) used to solve structure: *SIR92* (Altomare *et al.*, 1994 ▶); program(s) used to refine structure: *CRYSTALS* (Betteridge *et al.*, 2003 ▶); molecular graphics: *CAMERON* (Watkin *et al.*, 1996 ▶); software used to prepare material for publication: *CRYSTALS*.

## Supplementary Material

Crystal structure: contains datablock(s) global, I. DOI: 10.1107/S1600536811026985/fj2411sup1.cif

Structure factors: contains datablock(s) I. DOI: 10.1107/S1600536811026985/fj2411Isup2.hkl

Supplementary material file. DOI: 10.1107/S1600536811026985/fj2411Isup3.cml

Additional supplementary materials:  crystallographic information; 3D view; checkCIF report

## Figures and Tables

**Table 1:** Hydrogen-bond geometry (Å, °) *Cg*3 is he centroid of the C11–C16 ring.

*D*—H⋯*A*	*D*—H	H⋯*A*	*D*⋯*A*	*D*—H⋯*A*
C12—H12⋯*Cg*3^i^	0.97	2.86	3.630 (2)	136

Symmetry code: (i) 
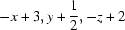
.

**Fig. 1.**
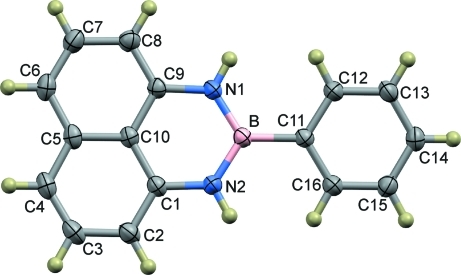
The title compound, showing the fll atom labelling scheme, with displacement ellipsoids drawn at the 50% probability level. H atoms are shown as spheres of arbitary radius.

**Fig. 2.**
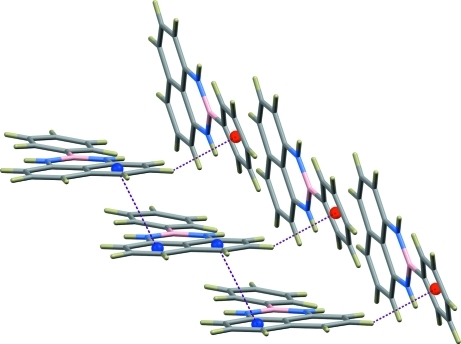
Intermolecular interactions of the title compound. The columns are supported by π-stacking and C–H···π interqactions. The resulting column runs collinear to the b axis.

## supplementary crystallographic information

### Comment

The title compound is approaching planarity. The biggest deviation from planarity lies in the phenyl ring, which subtends an angle of 9.0 (5)° ° relative to the naphthalene rings and the boron-nitrogen heterocycle. The N1—B—C11—C12 torsion angle is 6.0 (3) ° and the N2—B—C11—C16 torsion angle is 5.6 (3)°. The two N—B bonds are approximately equal (averaged to 1.414 (5) Å), the B—C11 bond length is 1.562 (2) Å. The N1—B—N2 bond angle measures 115.6 (2) ° while the N1—B—C11 and N2—B—C11 bond angles measure 122.0 (2) and 122.4 (2) °, respectively. These bond lengths and angles are in good agreement with those reported for structurally related diazaborolyl systems (Weber *et al.*).

The structure shows the molecules to be packed into infinite one-dimensional columns, supported by π···π interactions. The *Cg*1···*Cg*2 distance is 3.92 Å, where *Cg*1 and *Cg*2 are the centroids of two naphthyl rings of adjacent molecules. The one-dimensional chains run collinear with the *b* axis. The spacing between the mean planes of the naphthalene rings of two neighbouring molecules is 3.44 (4) Å. Two adjoining π—stacked columns are linked together by a C—H···π interaction between atoms C12–H12 and *Cg*3, where *Cg*3 is the centroid of the phenyl ring. The H12···*Cg*3 and C12···*Cg*3 distances are 2.86 (3)Å and 3.63 (3) Å, respectively, with a C12—H12 bond length of 0.970 (2) Å. The symmetry code is 2 - *x*, &frac12;+*y*, 2 - *z*.

### Experimental

To a solution of 1,8-diaminonaphthalene in toluene (4.11 mmol in 50 ml, 0.82*M*) (Letsinger, 1958, Slabber, 2011) was added the phenylboronic acid (4.11 mmol) in one portion. The round-bottomed flask was equipped with a Dean and Stark trap, and the solution was stirred and heated at 110°C for 3 h. The solvent was removed *in vacuo* and column chromatography of the crude solid on silica eluting with CH2Cl2 yielded pale green crystalline material in a yield of 77%. Crystals suitable for X-ray diffraction analysis were grown from CH_2_Cl_2_ at room temperature.

### Refinement

In the absence of significant anomalous scattering, Friedel pairs were merged.

The absolute configuration was arbitrarily assigned.

The relatively large ratio of minimum to maximum corrections applied in the multiscan process (1:nnn) reflect changes in the illuminated volume of the crystal.

Changes in illuminated volume were kept to a minimum, and were taken into account (Görbitz, 1999) by the multi-scan inter-frame scaling (*DENZO*/*SCALEPACK*, Otwinowski & Minor, 1997).

The H atoms were all located in a difference map, but those attached to carbon atoms were repositioned geometrically. The H atoms were initially refined with soft restraints on the bond lengths and angles to regularize their geometry (C—H in the range 0.93–0.98, N—H in the range 0.86–0.89 N—H to 0.86 O—H = 0.82 Å) and *U*_iso_(H) (in the range 1.2–1.5 times *U*_eq_ of the parent atom), after which the positions were refined with riding constraints.

### Crystal data

**Table:**

C_16_H_13_BN_2_	*F*(000) = 256
*M**_r_* = 244.10	*D*_x_ = 1.302 Mg m^−^^3^
Monoclinic, *P*2_1_	Mo *K*α radiation, λ = 0.71073 Å
Hall symbol: P 2yb	Cell parameters from 3114 reflections
*a* = 11.0117 (7) Å	θ = 3.5–32.1°
*b* = 5.4299 (2) Å	µ = 0.08 mm^−^^1^
*c* = 11.7454 (7) Å	*T* = 298 K
β = 117.574 (8)°	Amorphous, colourless
*V* = 622.52 (7) Å^3^	0.45 × 0.35 × 0.35 mm
*Z* = 2	

### Data collection

**Table:**

Oxford Diffraction Xcalibur 2 CCD diffractometer	1601 reflections with *I* > 2σ(*I*)
graphite	*R*_int_ = 0.025
ω/2θ scans	θ_max_ = 32.2°, θ_min_ = 3.5°
Absorption correction: multi-scan (*CrysAlis RED*; Oxford Diffraction, 2002)	*h* = −16→16
*T*_min_ = 0.966, *T*_max_ = 0.974	*k* = −6→7
6492 measured reflections	*l* = −17→17
2177 independent reflections	

### Refinement

**Table:**

Refinement on *F*^2^	Hydrogen site location: inferred from neighbouring sites
Least-squares matrix: full	H-atom parameters constrained
*R*[*F*^2^ > 2σ(*F*^2^)] = 0.039	Method = Modified Sheldrick *w* = 1/[σ^2^(*F*^2^) + (0.09*P*)^2^ + 0.01*P*], where *P* = [max(*F*_o_^2^,0) + 2*F*_c_^2^]/3
*wR*(*F*^2^) = 0.121	(Δ/σ)_max_ = 0.000415
*S* = 0.91	Δρ_max_ = 0.20 e Å^−^^3^
2173 reflections	Δρ_min_ = −0.16 e Å^−^^3^
173 parameters	Extinction correction: Larson (1970), Equation 22
1 restraint	Extinction coefficient: 140 (30)
Primary atom site location: structure-invariant direct methods	

### Fractional atomic coordinates and isotropic or equivalent isotropic displacement parameters (Å^2^)

**Table:**

	*x*	*y*	*z*	*U*_iso_*/*U*_eq_	
N1	1.07937 (13)	0.0122 (3)	0.90876 (12)	0.0456	
C9	0.94717 (15)	0.0472 (3)	0.80805 (13)	0.0417	
C10	0.92753 (16)	0.2405 (3)	0.71948 (14)	0.0406	
C1	1.03776 (16)	0.3948 (3)	0.73368 (14)	0.0430	
N2	1.16544 (14)	0.3539 (3)	0.83909 (13)	0.0491	
B	1.19265 (18)	0.1616 (4)	0.92917 (17)	0.0403	
C11	1.33755 (15)	0.1227 (3)	1.04553 (14)	0.0399	
C12	1.44379 (16)	0.2892 (3)	1.07293 (15)	0.0477	
C13	1.57062 (17)	0.2611 (4)	1.17915 (16)	0.0531	
C14	1.59475 (16)	0.0640 (4)	1.26062 (15)	0.0511	
C15	1.49189 (18)	−0.1046 (4)	1.23488 (17)	0.0560	
C16	1.36508 (17)	−0.0755 (3)	1.12917 (17)	0.0517	
C2	1.01683 (19)	0.5787 (4)	0.64568 (17)	0.0543	
C3	0.8862 (2)	0.6115 (4)	0.54197 (17)	0.0612	
C4	0.7787 (2)	0.4684 (4)	0.52663 (17)	0.0583	
C5	0.79515 (16)	0.2780 (4)	0.61455 (14)	0.0475	
C6	0.68699 (17)	0.1231 (4)	0.60321 (17)	0.0561	
C7	0.70890 (17)	−0.0588 (4)	0.68964 (17)	0.0580	
C8	0.83880 (17)	−0.0999 (4)	0.79292 (16)	0.0522	
H12	1.4293	0.4288	1.0166	0.0546*	
H13	1.6423	0.3824	1.1956	0.0605*	
H14	1.6813	0.0443	1.3339	0.0599*	
H15	1.5087	−0.2397	1.2902	0.0629*	
H16	1.2954	−0.1980	1.1133	0.0628*	
H2	1.0918	0.6844	0.6551	0.0617*	
H3	0.8736	0.7378	0.4805	0.0712*	
H4	0.6920	0.4919	0.4565	0.0616*	
H6	0.5982	0.1487	0.5315	0.0599*	
H7	0.6355	−0.1652	0.6800	0.0684*	
H8	0.8547	−0.2320	0.8529	0.0600*	
H101	1.0917	−0.1139	0.9595	0.0510*	
H102	1.2340	0.4503	0.8471	0.0527*	

### Atomic displacement parameters (Å^2^)

**Table:**

	*U*^11^	*U*^22^	*U*^33^	*U*^12^	*U*^13^	*U*^23^
N1	0.0460 (7)	0.0460 (7)	0.0388 (6)	−0.0010 (6)	0.0146 (5)	0.0082 (6)
C9	0.0420 (7)	0.0475 (9)	0.0358 (7)	0.0013 (7)	0.0182 (6)	−0.0005 (7)
C10	0.0424 (7)	0.0452 (9)	0.0354 (6)	0.0063 (7)	0.0190 (6)	0.0001 (6)
C1	0.0453 (8)	0.0455 (8)	0.0400 (7)	0.0084 (7)	0.0212 (6)	0.0054 (7)
N2	0.0402 (6)	0.0529 (9)	0.0506 (7)	0.0006 (6)	0.0180 (6)	0.0130 (6)
B	0.0415 (8)	0.0417 (9)	0.0387 (8)	0.0033 (7)	0.0194 (6)	0.0029 (7)
C11	0.0411 (7)	0.0403 (8)	0.0391 (7)	0.0041 (6)	0.0193 (6)	0.0031 (6)
C12	0.0479 (8)	0.0481 (9)	0.0445 (7)	0.0000 (8)	0.0191 (6)	0.0051 (8)
C13	0.0450 (8)	0.0589 (11)	0.0517 (9)	−0.0047 (8)	0.0191 (7)	−0.0027 (8)
C14	0.0421 (8)	0.0626 (11)	0.0430 (7)	0.0078 (8)	0.0150 (6)	0.0010 (8)
C15	0.0526 (9)	0.0582 (11)	0.0528 (9)	0.0112 (9)	0.0206 (7)	0.0179 (9)
C16	0.0452 (8)	0.0493 (10)	0.0555 (9)	0.0015 (8)	0.0190 (7)	0.0128 (8)
C2	0.0590 (9)	0.0540 (10)	0.0541 (9)	0.0104 (8)	0.0297 (8)	0.0142 (8)
C3	0.0716 (11)	0.0607 (11)	0.0490 (9)	0.0188 (10)	0.0260 (8)	0.0179 (9)
C4	0.0565 (9)	0.0651 (12)	0.0421 (8)	0.0178 (9)	0.0134 (7)	0.0059 (8)
C5	0.0463 (8)	0.0558 (10)	0.0374 (7)	0.0110 (8)	0.0168 (6)	−0.0022 (7)
C6	0.0422 (8)	0.0732 (12)	0.0455 (8)	0.0055 (9)	0.0141 (6)	−0.0074 (9)
C7	0.0454 (8)	0.0729 (13)	0.0548 (9)	−0.0082 (9)	0.0225 (7)	−0.0070 (9)
C8	0.0495 (9)	0.0610 (11)	0.0488 (8)	−0.0036 (8)	0.0250 (7)	0.0012 (8)

### Geometric parameters (Å, °)

**Table:**

N1—C9	1.4001 (18)	C14—C15	1.376 (3)
N1—B	1.412 (2)	C14—H14	0.949
N1—H101	0.876	C15—C16	1.383 (2)
C9—C10	1.422 (2)	C15—H15	0.940
C9—C8	1.377 (2)	C16—H16	0.966
C10—C1	1.420 (2)	C2—C3	1.401 (2)
C10—C5	1.422 (2)	C2—H2	0.969
C1—N2	1.3956 (19)	C3—C4	1.356 (3)
C1—C2	1.378 (2)	C3—H3	0.958
N2—B	1.416 (2)	C4—C5	1.412 (3)
N2—H102	0.888	C4—H4	0.937
B—C11	1.562 (2)	C5—C6	1.413 (3)
C11—C12	1.393 (2)	C6—C7	1.356 (3)
C11—C16	1.393 (2)	C6—H6	0.961
C12—C13	1.385 (2)	C7—C8	1.400 (2)
C12—H12	0.969	C7—H7	0.956
C13—C14	1.377 (3)	C8—H8	0.963
C13—H13	0.976		
			
C9—N1—B	123.84 (13)	C15—C14—H14	120.2
C9—N1—H101	117.0	C14—C15—C16	120.50 (17)
B—N1—H101	119.1	C14—C15—H15	119.3
N1—C9—C10	117.69 (13)	C16—C15—H15	120.2
N1—C9—C8	122.02 (14)	C11—C16—C15	121.42 (16)
C10—C9—C8	120.29 (14)	C11—C16—H16	120.1
C9—C10—C1	121.16 (12)	C15—C16—H16	118.4
C9—C10—C5	119.17 (14)	C1—C2—C3	119.49 (18)
C1—C10—C5	119.67 (14)	C1—C2—H2	120.4
C10—C1—N2	117.75 (13)	C3—C2—H2	120.1
C10—C1—C2	120.18 (14)	C2—C3—C4	121.63 (18)
N2—C1—C2	122.08 (15)	C2—C3—H3	118.6
C1—N2—B	123.93 (14)	C4—C3—H3	119.8
C1—N2—H102	117.3	C3—C4—C5	120.91 (16)
B—N2—H102	118.7	C3—C4—H4	120.7
N2—B—N1	115.57 (13)	C5—C4—H4	118.4
N2—B—C11	122.03 (14)	C10—C5—C4	118.13 (16)
N1—B—C11	122.37 (13)	C10—C5—C6	118.52 (16)
B—C11—C12	121.53 (14)	C4—C5—C6	123.35 (16)
B—C11—C16	121.48 (14)	C5—C6—C7	120.87 (16)
C12—C11—C16	116.95 (14)	C5—C6—H6	117.9
C11—C12—C13	121.70 (16)	C7—C6—H6	121.2
C11—C12—H12	119.6	C6—C7—C8	121.40 (17)
C13—C12—H12	118.7	C6—C7—H7	120.3
C12—C13—C14	120.09 (17)	C8—C7—H7	118.3
C12—C13—H13	119.3	C7—C8—C9	119.74 (17)
C14—C13—H13	120.6	C7—C8—H8	121.3
C13—C14—C15	119.32 (15)	C9—C8—H8	119.0
C13—C14—H14	120.5		

### Hydrogen-bond geometry (°)

**Table:**

Cg3 is he centroid of the C11–C16 ring.

**Table:**

*D*—H···*A*
—···
